# Host plant resistance for fall armyworm management in maize: relevance, status and prospects in Africa and Asia

**DOI:** 10.1007/s00122-022-04073-4

**Published:** 2022-03-23

**Authors:** Boddupalli M. Prasanna, Anani Bruce, Yoseph Beyene, Dan Makumbi, Manje Gowda, Muhammad Asim, Samuel Martinelli, Graham P. Head, Srinivas Parimi

**Affiliations:** 1 International Maize and Wheat Improvement Center (CIMMYT), ICRAF Campus, UN Avenue, P.O. Box 1041, GigiriNairobi, 00621 Kenya; 2 Bayer Pakistan (Private) Ltd, Karachi, Pakistan; 3 Regulatory Science, Bayer Crop Science US, Chesterfield, MO USA; 4 Bayer (South East Asia) Private Ltd, Singapore, Singapore

## Abstract

**Key message:**

Sustainable control of fall armyworm (FAW) requires implementation of effective integrated pest management (IPM) strategies, with host plant resistance as a key component. Significant opportunities exist for developing and deploying elite maize cultivars with native genetic resistance and/or transgenic resistance for FAW control in both Africa and Asia.

**Abstract:**

The fall armyworm [*Spodoptera frugiperda* (J.E. Smith); FAW] has emerged as a serious pest since 2016 in Africa, and since 2018 in Asia, affecting the food security and livelihoods of millions of smallholder farmers, especially those growing maize. Sustainable control of FAW requires implementation of integrated pest management strategies, in which host plant resistance is one of the key components. Significant strides have been made in breeding elite maize lines and hybrids with native genetic resistance to FAW in Africa, based on the strong foundation of insect-resistant tropical germplasm developed at the International Maize and Wheat Improvement Center, Mexico. These efforts are further intensified to develop and deploy elite maize cultivars with native FAW tolerance/resistance and farmer-preferred traits suitable for diverse agro-ecologies in Africa and Asia. Independently, genetically modified *Bt* maize with resistance to FAW is already commercialized in South Africa, and in a few countries in Asia (Philippines and Vietnam), while efforts are being made to commercialize *Bt* maize events in additional countries in both Africa and Asia. In countries where *Bt* maize is commercialized, it is important to implement a robust insect resistance management strategy. Combinations of native genetic resistance and *Bt* maize also need to be explored as a path to more effective and sustainable host plant resistance options. We also highlight the critical gaps and priorities for host plant resistance research and development in maize, particularly in the context of sustainable FAW management in Africa and Asia.

## Introduction

The fall armyworm (FAW), *Spodoptera frugiperda* (Lepidoptera: Noctuidae), is a migratory and highly polyphagous pest native to the Americas (Spark 1979). It is the most economically important pest of maize in South and Central America (Cruz et al. 2012; Nagoshi et al. 2014, 2015). Due to the absence of a diapause trait, in the USA, it migrates northward annually in the fall season to infest temperate maize cropping areas (Westbrook et al. 2016). FAW comprises two genetically and behaviorally separate strains that occur sympatrically throughout North and South America (Pashley 1986). The “corn” strain (C) was reported to be preferentially damaging maize, sorghum and cotton, whereas the “rice” strain (R) predominantly infests rice, alfalfa, pasture and forage grasses (Nagoshi et al. 2007; Juarez et al. 2012). These “host strains” of FAW are morphologically indistinguishable; however, polymorphisms in the mitochondrial cytochrome oxidase subunit I (COI) gene proved to be capable of consistently differentiating and identifying the C and R strains based on their haplotypes (Levy et al. 2002).

FAW was officially reported outside the Americas for the first time in West Africa in January 2016 (Goergen et al. 2016), and by January 2018, the pest was reported in over 40 African countries (Prasanna et al. 2018). In Asia, FAW was first reported in the southern Indian state of Karnataka in May 2018 (Sharanabasappa et al. 2018; Shylesha et al. 2018), and subsequently in all the maize-growing states in the country (Suby et al. 2020; Deshmukh et al. 2021). Between 2018 and 2021, the pest rapidly spread across the Asia–Pacific region and has been reported from Yemen, Bangladesh, Myanmar, China, Thailand, Sri Lanka, Nepal, Philippines, Vietnam, Indonesia, Australia, South Korea, Cambodia, Papua New Guinea, Timor Leste, New Caledonia, Jordan, Syria and the United Arab Emirates (reviewed by Prasanna et al. 2021a).

The rapid spread and establishment of FAW populations in Africa and Asia highlight two important facts: a) similar to the Americas, the pest can spread quickly across large geographic areas within a short timeframe (Prasanna et al. 2021a, b) FAW populations can persist throughout the year in the conducive tropical/subtropical climates. Thus, FAW has now become a global problem, posing a serious threat to the food and nutritional security and livelihoods of hundreds of millions of farming households, especially those dependent on maize, in both Africa and Asia. Although FAW incidence has been reported on several crops, including maize (field/sweet/waxy), sorghum, sugarcane, wheat, rice (very limited), millets, ginger, soybean, tomato, cotton, cabbage, groundnut, banana, pasture grasses and green amaranth, the pest has caused major economic damage mostly to maize across Africa and Asia, and secondarily to sorghum and sugarcane (to a limited extent). The economic impact of FAW is not only represented by the extensive yield losses caused by the pest to the affected crops like maize (Rwomushana et al. 2018; Matova et al. 2020), but also additional management costs incurred by smallholder farmers—for example, the cost of managing the pest using various technologies, especially synthetic pesticides, besides increased labor. A recent study (Yang et al. 2021) examining the response of farmers to FAW in Yunnan province in China showed that the full cost of pesticide-based crop protection increased from US$81 per hectare per crop season in 2018 to US$276 in 2020, mainly due to FAW. The study also showed that at the FAW infestation levels present, some farmers were applying, on average, as many as 6.4 pesticide applications per crop season in 2020. We still do not know the magnitude of environmental cost of such extensive pesticide applications that could potentially affect natural enemies of insect pests.

There is no single solution that can provide sustainable control of a complex pest like FAW. A wide range of technologies and management practices have been developed over the years and are available for control of FAW, including host plant resistance, cultural control, biological control, biopesticides, mating disruption technologies, synthetic pesticides and agroecological management (Prasanna et al. 2018, 2021b). However, these technologies are not equally accessible to farming communities in countries across Africa or Asia due to various reasons, including regulatory bottlenecks. More generally, efficacy, cost/affordability, safety, accessibility and scalability are all important factors to consider in assessing the potential combination of technologies or management practices for IPM of FAW. In this review, we focus on the status of host plant resistance—both native traits and transgenic FAW resistance—for FAW control in Africa and Asia, as well as future opportunities and critical needs in host plant resistance research and development.

## Relevance of host plant resistance in FAW management

“Host plant resistance,” in the context of resistance to insect pests, is defined as “the collective heritable characteristics by which a plant species may reduce the probability of successful utilization of that plant as a host by an insect species” (Beck 1965). Host plant resistance is a central component of IPM strategies to control FAW (Prasanna et al. 2018, 2021c) and comprises: a) native genetic resistance: identifying/developing germplasm with resistance to FAW, and b) transgenic resistance: using a gene or combination of genes from an external source(s) (other than the recipient plant) to make the host plant resistant to FAW. FAW-tolerant/FAW-resistant varieties, whether derived through native genetic resistance or through a transgenic approach, provide a practical and economical way to minimize crop losses due to the pest. In addition, improved maize varieties with genetic resistance to FAW effectively complement other IPM interventions (Riggin et al. 1992, 1994). Seed-based technologies like host plant resistance are not only easily disseminated and readily adopted by farmers due to their visible benefits, but also require fewer applications of pesticides than the FAW-susceptible varieties.

When designing a breeding strategy to introduce FAW resistance traits into elite maize germplasm, breeders consider not only the source and strength of FAW resistance, but also the potential durability of resistance over time. Insect pests such as FAW can evolve to overcome monogenic (based on a single gene) or oligogenic (based on a few genes) resistance, as has been observed with transgenic crop varieties (Huang et al. 2014). Breeding for FAW resistance is, therefore, a continuous process, with no “finish line” to the perpetual race between the host and the evolving pest. As a general principle, breeding programs should seek to identify, utilize, and ultimately combine multiple resistance traits—whether conventional or, where approved for use, transgenic—to improve the durability of host plant resistance (Prasanna et al. 2018, 2021c).

Prasanna et al. (2018, 2021c) presented a comprehensive review of host plant resistance to FAW, especially in maize. This included information on potential sources of resistance to FAW in tropical and temperate maize germplasm identified or developed earlier by maize breeding programs in the Americas, and detailed protocols for mass rearing of FAW and screening germplasm under artificial FAW infestation. Here we (a) provide an update on the progress made in breeding for native genetic resistance to FAW in maize; (b) highlight the status of deployment of genetically modified (GM) maize (specifically *Bt* maize) in Africa and Asia for the control of FAW, and the need to implement effective insect resistance management (IRM) strategies for *Bt* maize; and (c) suggest possible next steps for making host plant resistance an integral component of an IPM-based strategy for sustainable management of FAW in sub-Saharan Africa (SSA) and Asia.

## Breeding for native genetic resistance to FAW

### FAW tolerance in tropical maize germplasm

CIMMYT has a wealth of diverse genetic resources in maize, including landrace accessions as well as improved germplasm with an array of traits (e.g., high yield, drought tolerance, heat tolerance, nitrogen use efficiency, and resistance to various diseases and insect-pests) that are relevant for the smallholders. The maize germplasm bank at CIMMYT, Mexico, holds nearly 27,000 accessions that provide a rich platform for identifying genetic resources for various farmer-preferred traits. Research conducted at CIMMYT in Mexico (Mihm 1997) and subsequently in Africa and Asia revealed that there are genetic variation and potential to support breeding for native genetic resistance to insect pests of maize, including stem borers (e.g., spotted stem borer, *Chilo partellus*; maize stalk borer, *Busseola fusca*; Asian corn borer, *Ostrinia furnicalis*; and European corn borer, *Ostrinia nubilalis*), FAW and post-harvest pests (weevils and large grain borer).

The work done at CIMMYT-Mexico led to the development of two major maize populations—Multiple Insect-Resistant Tropical (MIRT) and Multiple Borer Resistant (MBR)—that served as the foundation for deriving improved tropical/subtropical maize inbred lines with at least partial resistance to FAW. These insect-resistant maize populations were derived primarily from the Caribbean maize germplasm and Tuxpeño landrace accessions from Mexico (Mihm 1997). CIMMYT and partners in Africa have utilized the insect-resistant maize populations and inbred lines from Mexico and developed elite maize germplasm (hybrids and improved open-pollinated varieties or OPVs) with resistance to other lepidopteran stem borers, including the European corn borer (*Ostrinia nubilalis* Hbn.), the African stem borer (*Busseola fusca* Fuller) and the spotted stem borer (*Chilo partellus* Swinhoe) (Murenga et al. 2015; Tefera et al. 2016a, b). Some of these insect-resistant materials have the potential to offer resistance against FAW, which is also a lepidopteran pest.

**Fig. 1:**
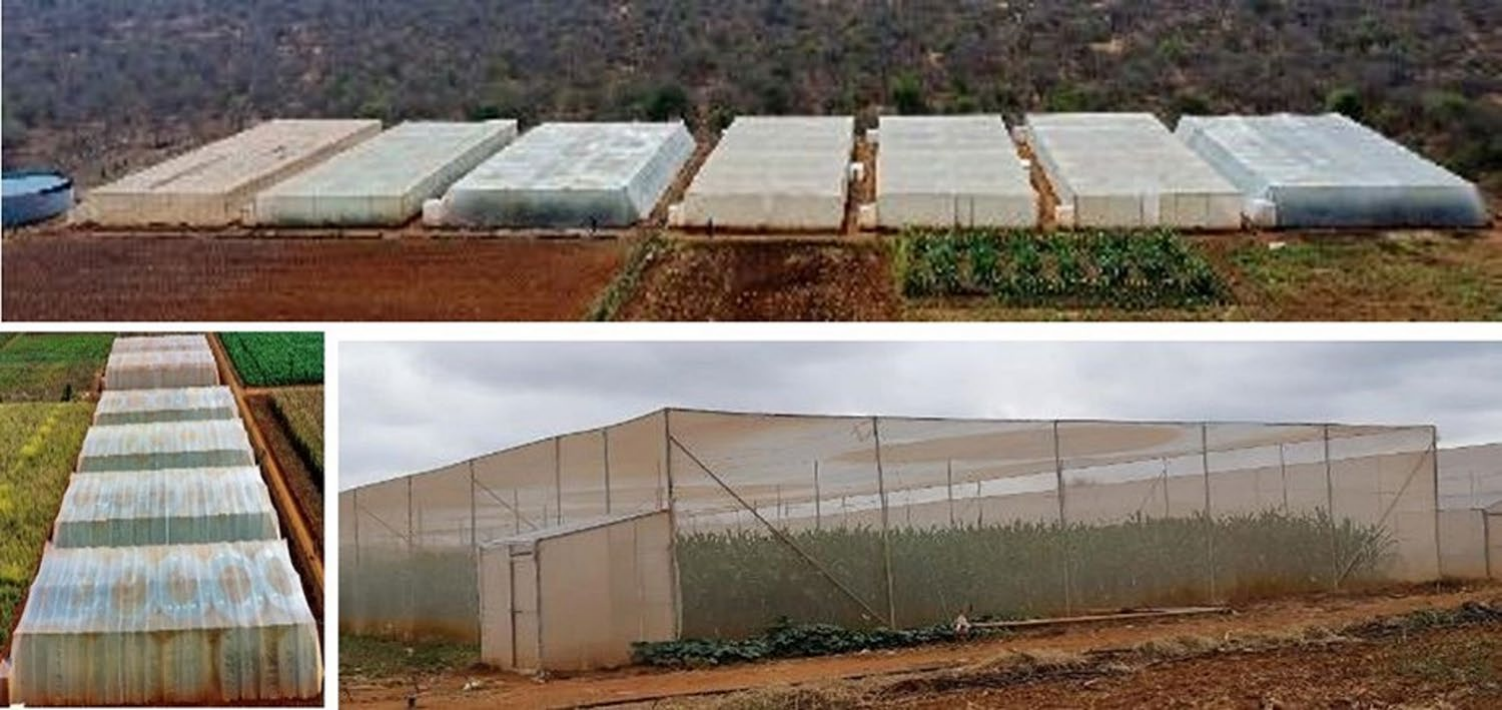
Germplasm screening under FAW artificial infestation in screenhouses is critical in breeding for native genetic resistance to FAW. The figure shows the screenhouse complex established by CIMMYT at KALRO Research Center at Kiboko, Kenya

A screenhouse complex (with 13 screenhouses, each 1000 m^2^) was established by CIMMYT at Kenya Agricultural and Livestock Research Organization (KALRO) Research Center in Kiboko, Kenya, in 2017–2018, for intensive screening of maize germplasm against FAW under artificial infestation and for identifying and developing promising FAW-tolerant inbred lines and hybrids (Fig. 1). Each screenhouse can accommodate 245 maize rows of 3 m length with 12 plants per row. A similar screenhouse facility has been established by CIMMYT at Hyderabad, India. When identifying materials with native genetic resistance to FAW, it is important to consider not only foliar damage but also ear/cob damage, as FAW larvae can cause significant ear/kernel damage by entering the developing ears. CIMMYT uses a 1–9 foliar damage scale (Prasanna et al. 2018), which is a modification of the Davis et al. (1992) 0–9 scale, for assessing the responses of maize germplasm against FAW under artificial infestation. At physiological maturity (harvest), the CIMMYT team also evaluates the maize germplasm on a 1–9 ear damage scale, as described by Prasanna et al. (2018). In addition, other parameters, including percentage ear rot and number of exit holes per ear, are recorded. The cumulative foliar and ear damage scores, along with grain yield and other parameters, are considered for final rating of the germplasm and for taking selection decisions.

Starting in 2017, the CIMMYT maize breeding program in Kenya implemented intensive efforts to identify and develop maize germplasm with tolerance/resistance to FAW. FAW-tolerant maize germplasm developed earlier at CIMMYT-Mexico, as well as inbred lines, OPVs and hybrids developed by CIMMYT in Africa through the Insect-Resistant Maize for Africa (IRMA) project (Tefera et al. 2016a; Prasanna et al. 2018), were screened. Between 2017 and 2020, over 6000 maize genotypes, including inbred lines and hybrids/OPVs from diverse sources, were screened under FAW artificial infestation in the screenhouses at Kiboko. The work led to identification/validation of promising FAW-tolerant/FAW-resistant inbred lines, especially from the MBR and MIRT germplasm backgrounds, with low foliar and ear damage scores.

### FAW-tolerant/FAW-resistant CIMMYT maize inbred lines

Several FAW-tolerant/FAW-resistant CIMMYT maize inbred lines have been developed and disseminated to interested institutions globally over the years. These inbred lines include lines identified earlier at CIMMYT-Mexico and validated under artificial infestation at CIMMYT-Kenya (e.g., CML71, CML124, CML125, CML333, CML334, CML338), as well as some of the CIMMYT maize lines that were found to offer resistance to FAW in Kenya (e.g., CML370, CML372 and CML574). Since 2018, FAW-tolerant/FAW-resistant CMLs have been disseminated to 92 institutions in 34 countries globally, including an array of NARES, advanced research institutes (ARIs) and commercial seed companies in the Americas, Europe, Africa, Asia and Australia (Table 1). These CMLs^1^ can be potentially utilized as trait donors in breeding programs of institutions that are developing FAW-tolerant maize cultivars for local environments.

**Table 1:** Global dissemination of CIMMYT’s FAW-tolerant/FAW-resistant CMLs (since 2018)

Type of institution	Africa	Asia	Latin America	North America*	Europe	Australia	Total
NARES/ARIs/Universities	14 (11)	9 (6)	14 (5)	3 (2)	1 (1)	2 (1)	**43 (26)**
Commercial seed companies	11 (7)	10 (6)	22 (4)	2 (1)	4 (3)	-	**49 (21)**
**Total**	**25 (13)**	**19 (9)**	**36 (6)**	**5 (2)**	**5 (3)**	**2 (1)**	**92 (34)**

Figures in parentheses indicate number of countries

*Mexico is included under Latin America

Several national maize breeding programs in Africa and Asia have initiated breeding programs for developing FAW-tolerant cultivars (e.g., Matova et al. 2020; Kasoma et al. 2020), especially utilizing sources of native genetic resistance from CIMMYT. Besides the CMLs mentioned above, the CIMMYT team in Africa has identified over the last two years several promising inbred lines in both yellow-and white-kernel backgrounds, with tolerance/resistance to FAW, based on low foliar and ear damage and competitive grain yields. For example, crosses were made among the promising lines, from which progenies were selected and intercrossed to increase the frequency of resistance alleles. Doubled haploid (DH) lines were developed from F_1_, F_2_ and backcross (BC) source populations with resistance to FAW. In 2019–2020, a total of 2733 DH lines were produced from different source populations. From these, a set of 1400 DH lines were screened under FAW artificial infestation at the screenhouses in Kiboko, leading to identification of new FAW-resistant lines. These lines are used to make new single-crosses and three-way hybrids for further evaluation.

### Development of elite FAW-tolerant tropical maize hybrids

Based on the results from screening of a large collection of inbred lines from different genetic backgrounds during 2017–2018, the CIMMYT team in Kenya formed single-cross and three-way-cross hybrids. In 2018, a set of 197 single-cross hybrids were developed and evaluated under artificial FAW infestation. The best FAW-tolerant/FAW-resistant single-crosses have been used (a) as female parents to develop three-way hybrids, (b) to make narrow-based synthetics, and (c) as source populations for DH induction to develop new FAW-resistant lines. In 2019, 88 three-way hybrids showed genetic variation for grain yield under various conditions and FAW damage parameters. Hybrids with MBR and MIRT backgrounds were among those that showed a combination of low ear damage and good grain yield across various conditions. In 2019–2020, over 500 hybrids, including single- and three-way crosses, were tested across different management conditions, including screening against FAW under artificial infestation in Kiboko. Stage gate advancement of promising maize hybrids with native genetic resistance is implemented by considering both foliar damage and ear damage scores below specific thresholds (≤ 5.0 and < 3.0, respectively), in addition to significantly higher grain yield than FAW-susceptible commercial checks (WE3106 and DK777).

**Fig. 2:**
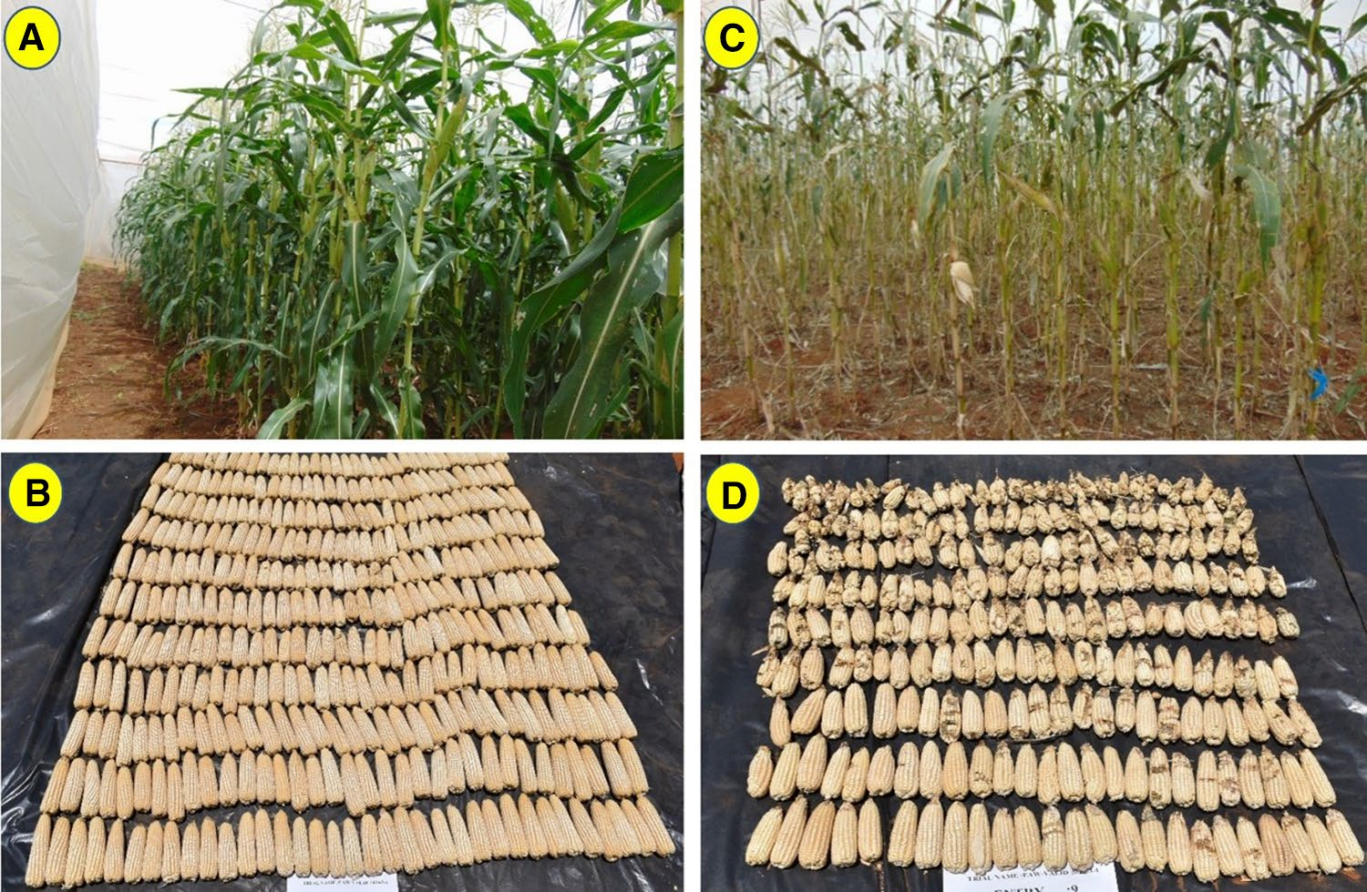
“No-choice trial” of one of the promising FAW-tolerant maize hybrids versus a FAW-susceptible commercial hybrid under FAW artificial infestation in Kiboko, Kenya in 2020. Note the distinct differences in terms of foliar damage of FAW-tolerant (**A**) versus FAW-susceptible hybrid (**C**) and the differences in ear yield and ear damage (visible as blackish spots with no grains in the ears) of the FAW-tolerant hybrid (**B**) versus susceptible hybrid (**D**)

Based on the results of on-station screenhouse trials against FAW in Kiboko during 2017–2019, the CIMMYT maize team in Africa further evaluated in 2020 a set of eight promising white-grained hybrids (four early-maturing and four intermediate-maturing) against four widely used commercial hybrids (two early-and two intermediate-maturing) as checks under different management conditions. Of particular importance are “no-choice trials” under FAW artificial infestation in screenhouses in Kiboko, Kenya (Fig. 2), which provide a clear assessment of the ability of the genotype to “resist” the pest and provide acceptable yield. In this trial, each entry was planted in 40 rows in a separate screenhouse compartment (“no-choice”) and each plant was infested with seven FAW neonates 14 days after planting. Foliar damage was assessed 7, 14, and 21 days after infestation, followed by ear damage and grain yield at harvest.

In addition, promising entries were also evaluated for their performance under managed drought stress, managed low-nitrogen stress and for their responses to key diseases (Turcicum leaf blight, Maize streak virus, Maize lethal necrosis, ear rots, etc.). The hybrids and their parents were also characterized on-station for their seed production characteristics, including maximum flowering time difference between parents and single-cross female parent seed yield. Besides on-station trials, the test hybrids along with commercial checks were evaluated under farmers’ management conditions (without any insecticide spray) at 16 on-farm sites in Kenya. Each entry was planted in 20-row plots and data were recorded on natural FAW infestation. Foliar damage was assessed 7, 14, 21, 28 and 35 days after germination together with insect incidence. Ear damage and percent ear damage were also recorded, as well as grain yield and other agronomic parameters. Based on data collected from the on-station and on-farm trials, three promising FAW-tolerant elite maize hybrids were announced by CIMMYT in December 2020 (https://www.cimmyt.org/news/announcing-cimmyt-derived-fall-armyworm-tolerant-elite-maize-hybrids-for-eastern-and-southern-africa/) for partners, especially in SSA. National performance trials (NPTs) of the three FAW-tolerant elite maize hybrids are presently undertaken by several countries in SSA.

### FAW resistance in temperate maize germplasm

The USDA-ARS Corn Host Plant Resistance Research Unit (CHPRRU) in Mississippi, USA (https://www.ars.usda.gov/southeast-area/mississippi-state-ms/crop-science-research-laboratory/corn-host-plant-resistance-research/) has a long history of conducting research on native genetic resistance to FAW, especially with temperate maize germplasm. During the late 1980s and throughout the 1990s, USDA-ARS and CIMMYT (Mexico) collaborated extensively on developing FAW-resistant maize germplasm in Mexico (Williams and Davis 1997). USDA-ARS researchers developed protocols for infesting maize plants with neonates and evaluating the resulting damage and thus identified temperate maize inbred lines with resistance to FAW (e.g., Mp705) (Prasanna et al. 2018). In addition to USDA-ARS, temperate maize germplasm with native genetic resistance to FAW has been developed by Embrapa-Brazil, University of Florida, USA, and the Germplasm Enhancement of Maize (GEM) project (Table 2).

**Table 2:** Some potential sources of FAW resistance in temperate maize germplasm identified or developed by maize breeding programs in the Americas

Germplasm	Description	References
Mp496; Mp701-Mp708, Mp713; Mp714; Mp716	Temperate maize inbred lines with resistance to FAW developed in the USA by USDA-ARS (Mississippi). Of these, Mp496, and Mp701 to Mp708, were derived from Caribbean accessions—Antigua Gp1, Antigua Gp2D, Guadalupe Gp1A, and Republica Dominica Gp1. Mp713 and Mp714 were developed from CIMMYT’s Multiple Insect-Resistant populations	Scott and Davis (1981); Scott et al. (1982); Williams and Davis (1980, 1982, 1984, 2000, 2002); Williams et al. (1990)
B49; B52; B64; B68; B96	Corn borer-tolerant inbreds generated by Iowa State University, USA, through introgression of Maiz Amargo from Argentina into temperate maize. The most important inbred line among these was B68, which became widely used by the seed industry in the USA	Walter Trevisan, personal communication
Three GEM (Germplasm Enhancement of Maize) inbreds, including XL370A	Derived from the introgression of germplasm from Uruguay, Cuba, and Thailand; showed resistance to FAW in the southern USA	Ni et al. (2014); Abel et al. (2020)
CMS14C; CMS23 (Antigua x Republica Dominica); CMS24; MIRT (Multiple Insect Resistance Tropical) race Zapalote Chico, Sintetico Spodoptera, Caatingueiro Spodoptera, and Assum Preto Spodoptera	Since 1975, Embrapa-Brazil identified and described several sources of resistance to FAW in maize, while also investigating the chemical compounds that underlie these native resistance traits	Walter Trevisan, personal communication
Brazilian maize lines with potential resistance to FAW	In work conducted from 1986 to 1993, Embrapa-Brazil identified potential sources of resistance to FAW based on evaluation of maize accessions in the Brazilian germplasm bank	Viana and Guimares (1997)

*Source*: Modified based on Prasanna et al. (2018)

## Genetic and molecular bases of native genetic resistance to FAW in maize

In the context of insect pests, “resistance” is the capacity to minimize feeding damage through mechanisms such as antibiosis and/or antixenosis, while “tolerance” is the ability to limit economic damage even in the presence of the pest (outside/inside the host). Native resistance in maize to FAW is polygenic and quantitative in nature, conferring partial resistance. The quantitative and polygenic nature of native genetic resistance is less vulnerable to resistance evolution in the targeted FAW populations than if native resistance was high level and or monogenic. Although we do not know comprehensively the genetic, biochemical and molecular bases of native genetic resistance in maize, some interesting insights are available and are summarized below.

Resistance to an insect pest may involve a combination of antibiosis, antixenosis, and/or tolerance (Painter 1958). Earlier studies evaluating FAW-resistant maize germplasm showed that the mechanisms could be quite varied, with some showing non-preference and others antixenosis (Wiseman et al. 1982). Non-preference could be due to distinct morphological traits (e.g., leaf thickness/toughness, very tight husk cover, kernel hardness) that minimize foliar or ear damage by FAW. Ni et al. (2008) showed that CML333 (with moderate silk maysin), CML336 (with low silk maysin) and CML338 (with high silk maysin) were resistant to FAW at the seedling stage, and CML335 (without silk maysin) was susceptible. CML338 and Mp708 were categorized as tolerant to insect herbivory because uninfested and injured plants showed no differences in photosynthetic rate, light response curves or photosynthetic rate in the A/Ci curves (net CO_2_ assimilation rate, A, versus calculated substomatal CO_2_ concentration, Ci).

Shivaji et al. (2010) analyzed whether constitutive levels of jasmonic acid (JA) and other octadecanoid compounds were elevated prior to herbivory in a maize genotype Mp708 with documented resistance to FAW and other lepidopteran pests. The inbred Mp708 had approximately threefold higher levels of JA prior to herbivore feeding than the susceptible inbred Tx601. In addition, the constitutive expression of JA-inducible genes, including those in the JA biosynthetic pathway, was higher in Mp708 than  in Tx601. In response to herbivory, Mp708 generated higher levels of hydrogen peroxide and NADPH oxidase transcripts before and after FAW larval feeding. The authors concluded that Mp708 could have a portion of its defense pathway primed resulting in constitutive defenses and the ability to mount a stronger defense when FAW larvae attack. Analysis of volatiles released by the FAW-resistant Mp708 and FAW-susceptible Tx601 in the presence and absence of FAW larvae led to the identification of (E)-β-caryophyllene, a terpenoid associated with FAW resistance, released constitutively in Mp708 (Smith et al. 2012). Israni et al. (2020) recently demonstrated that FAW utilizes specific UDP-Glycosyltransferases to detoxify or inactivate maize defensive benzoxazinoids.

Malook et al. (2021) identified a Chinese maize inbred line Xi502 that was able to mount effective defense in response to FAW attack. Comparative transcriptomics analyses, phytohormonal measurements and targeted benzoxazinoid quantification demonstrated significant inducible defense responses in Xi502 but not in the susceptible reference inbred line B73. The study also showed that Xi502 accumulates higher levels of benzoxazinoids effective against FAW than B73.

There is still a lot to learn about the genetic architecture of native genetic resistance to FAW in maize, although a few studies carried out in recent years have given some insights. Brooks et al. (2007) used 91 simple sequence repeat (SSR) markers on 213 F_2:3_ families and detected quantitative trait loci (QTLs) on chromosomes 1, 2, 6, 7, and 9. Womack et al. (2018) evaluated 231 F_2:3_ families from the cross of Mp704 (resistant) × Mo17 (susceptible) and genotyped with both SSR and single-nucleotide polymorphism (SNP) markers. This study revealed QTLs in chromosome bins 1.09, 2.08, 3.08, 6.02, 7.04, 8.03, 9.03, 10.02 and 10.04. Womack et al. (2020) developed a bi-parental mapping population, comprising 243 F_2:3_ families from the cross Mp705 (resistant) × Mp719 (susceptible), and evaluated this population for FAW leaf-feeding damage under artificial infestation over 3 years in the USA. QTL analyses led to identification of two major QTLs in bins 4.06 and 9.03 that together explained 35.7% of the phenotypic variance over all environments. The QTL identified in bin 9.03 co-located with a previously identified QTL associated with resistance to leaf-feeding damage in maize by FAW and other lepidopteran insects, while the QTL in bin 4.06 is a new source of resistance to FAW leaf-feeding damage identified in this study. Badji et al. (2020) evaluated a set of 316 tropical maize lines under natural FAW pressure in Uganda and identified 14 SNPs through genome-wide association study (GWAS). These SNPs are distributed on all chromosomes except chromosomes 6 and 7. Several FAW resistance QTLs discovered in earlier studies (Brooks et al. 2005, 2007; Womack et al. 2018) co-localized with 6 of the 14 SNPs reported by Badji et al. (2020).

Kamweru et al. (2022) recently undertook GWAS on a set of 423 CIMMYT maize lines to dissect the genetic basis of native resistance to FAW. The lines were evaluated, based on foliar and ear damage scores, against FAW under artificial infestation at Kiboko, Kenya, in 2020 and 2021. All the screened lines were genotyped with the DArTseq genotyping-by-sequencing platform (Diversity Arrays Technology/DArT). The study revealed 56 significant marker–trait associations and the predicted functions of the putative candidate genes varied from a defense response to several genes of unknown function. Chromosome 4 accounted for the highest number (15%) of the SNP markers associated with foliar damage. One major effect QTL on chromosome 9 in bin 9.03, reported in previous studies (Womack et al. 2018, 2020), coincided with SNP *DT9_96875821*, detected for the foliar damage score on the 14^th^ day after infestation. Another major QTL detected on bin 4.06 coincided with SNP *DT4_167218393*, detected for the foliar damage score 21 days after infestation. Another SNP (*DT8_165270110*) located on chromosome 8 contributed the strongest estimated effect size (6.50) for the expression of the leaf feeding damage resistance trait. Overall, the study revealed that native genetic resistance to FAW is quantitative in nature and is controlled by many loci with minor effects. Genomic selection/prediction could play an important role in improving native genetic resistance to FAW in maize.

## Transgenic resistance to FAW

### Development of *Bt* maize technologies and their use against FAW

The first GM crops developed in the late 1980s and 1990s expressed lepidopteran-active insecticidal proteins from *Bacillus thuringiensis* Berliner (and hence are known as *Bt* crops) (Fischhoff et al. 1987; Perlak et al. 1990). These proteins had been used previously in formulations of microbial products to control insect pests worldwide (US EPA 1998a, b; Huang et al. 2007; Hammond and Koch 2012). The efficacy and safety to humans and non-target organisms of these insecticidal proteins made them the optimal candidates for the development of GM crops (Koch et al. 2015; Romeis et al. 2019). By 2019, *Bt* crops expressing one or more *Bt* proteins for pest control were grown on 56 million hectares globally (ISAAA 2019), reflecting the economic and environmental benefits accrued by farmers due to the high efficacy of *Bt* crops (Carrière et al. 2003; Mendelsohn et al. 2003; Wu et al. 2008; Hutchinson et al. 2010; Dively et al. 2018) and their potential to reduce insecticide use (Zhang et al. 2018).

As a key lepidopteran maize pest in South America, *Bt* maize technologies have been successfully used to reduce FAW larval feeding injury in Brazil, Argentina, Paraguay and other major maize-growing countries (Fatoretto et al. 2017). Although the first generation of *Bt* maize technologies available in the region was based on the Cry1Ab protein, which has limited activity against FAW, these *Bt* maize hybrids still offered average protection against FAW that was superior to conventional insecticides. The behavior of FAW larvae to feed within the whorl of maize plants typically reduces the success of insecticidal sprays to manage FAW (Burtet et al. 2017). Moreover, resistance to various classes of synthetic insecticides including pyrethroids and organophosphates (Diez-Rodríguez and Omoto 2001; Carvalho et al. 2013), larval growth inhibitors (Nascimento et al. 2016), spinosyns (Okuma et al. 2018) and recently diamides (Bolzan et al. 2019; Boaventura et al. 2020) has been documented in FAW in Brazil. The highly destructive nature of FAW and the limited efficacy to insecticidal sprays favored the early adoption of *Bt* maize technologies by farmers in Brazil and surrounding countries. For instance, MON 810 hybrids containing Cry1Ab were commercially deployed in 2008 in Brazil and were perceived as an important contribution to more effective IPM for FAW (Waquil et al. 2013). Subsequently, the deployment of *Bt* maize hybrids expressing *Bt* proteins with significantly higher activity against FAW (Cry1F, Cry1A.105 and Cry2Ab2, and Vip3Aa20) led to higher adoption of *Bt* maize; *Bt* maize hybrids currently represent approximately 80% of the maize area in Brazil (Waquil et al. 2013; Marques et al. 2019; Moscardini et al. 2020). Extensive cultivation of *Bt* maize had no significant effect on non-target organisms, including parasitoids and predators of FAW (Comas et al. 2014; Resende et al. 2016; Bertho et al. 2020).

The same *Bt* maize technologies developed and commercialized for lepidopteran pest control in the Americas have been tested for their efficacy against key African and Asian maize pests to determine their fit for those regions. Where they promise good control of these pests, now including FAW, these technologies could be an important additional tool for farmers. However, a prerequisite for *Bt* maize technologies to be legally cultivated by farmers is assessment of the safety of the transgenic event to humans, non-target organisms and the environment and approval by national regulatory authorities. This in turn requires the presence of suitable regulatory frameworks and assessment capacity, which do not exist in many African and Asian countries. Where the capacity is present, regulatory approvals typically take many years and represent a significant barrier to the adoption of *Bt* maize. This situation contrasts with the simple and rapid process for registering hybrids with the sort of novel native traits described in the previous sections. Not surprisingly, the countries cultivating the largest areas of *Bt* crops globally typically have regulatory frameworks for *Bt* crops that are expeditious, transparent, and relatively simple to comprehend and comply with (Levin, 1994; ISAAA 2020; Turnbull et al. 2021). Because of the regulatory hurdles, political barriers and other factors, experience with *Bt* maize in Africa and Asia still is relatively limited, as summarized in the following sections.

### Bt maize in Africa

In South Africa, the first *Bt* maize hybrids (MON 810 with Cry1Ab) were planted commercially in 1997. In 2010, MON 89,034 hybrids with the Cry1A.105 and Cry2Ab2 proteins were introduced. These technologies were grown to control the stem borers, *Busseola fusca* (Lepidoptera: Noctuidae) and *Chilo partellus* (Lepidoptera: Crambidae). As in the Americas, these technologies have provided economic and environmental benefits to farmers, including regions comprised largely of smallholders (Keetch et al. 2005; Muzhinji and Ntuli 2020; Ala-Kokko et al. 2021). The area planted to GM maize in 2017 was estimated at 1.96 million ha, representing 85% of the total maize area (approximately 2.3 million ha) (FAOSTAT 2021). Of this area, 1.62 million ha was planted with *Bt* maize hybrids containing the Cry1Ab protein (MON 810) or the Cry1A.105 and Cry2Ab2 proteins (MON 89,034) (ISAAA 2017).

Since 2016, FAW has become a major pest in parts of the South African maize-growing area. After its invasion into South Africa in early 2016, FAW was included as a target pest of MON 89034 (Botha et al. 2019). Consistent with experience in the Americas, MON 89034 was observed to provide high levels of protection against FAW with superior yields in South Africa (Chingombe 2020). In contrast, the MON 810 maize event confers partial resistance to FAW.

South Africa is currently the only African country where *Bt* maize is grown commercially. However, the National Agricultural Research Organizations of Kenya, Ethiopia, Nigeria, Tanzania, Uganda and Mozambique are testing the performance of *Bt* maize technologies introgressed into locally adapted African maize hybrids under the TELA® Maize project (AATF 2021). The TELA maize project was launched in 2018 as a public–private partnership led by the African Agricultural Technology Foundation (AATF). The TELA maize project is working to enhance food security in sub-Saharan Africa (SSA) through the release of high yielding insect-protected (TELA®) maize hybrids.

### Bt maize in Asia

In Asia, experience with *Bt* maize has been broader than in Africa but is still limited. *Bt* maize technologies were first approved for cultivation in the Philippines in 2002 and Vietnam in 2014. The primary target pest for *Bt* maize in these countries is the Asian corn borer (*Ostrinia furnacalis* Guenée) (ACB), while secondary target pests include the common cutworm (*Spodoptera litura* Fabricius) (CCW), the corn earworm (*Helicoverpa armigera* Hübner) (CEW) and more recently FAW. *Bt* maize is also approved but not yet commercially planted in Pakistan, where the primary target pest is the maize stem borer (*Chilo partellus* Swinhoe). In addition, *Bt* maize is in the testing and approval process in China, the largest maize-growing country in Asia.

In the Philippines, *Bt* maize products have been cultivated since 2002 when the first *Bt* maize event, MON 810 (Yieldgard™), containing the Cry1Ab protein, was approved for commercial use. The approval of the first pyramided insect-resistant maize hybrid containing the MON 89034 event was received by Monsanto Philippines in 2010 (Table 3), followed by approval of Intrasect® hybrids from Pioneer Hi-Bred Philippines, containing the TC1507 × MON 810 events for insect resistance. The maize products containing the MON 810, Bt11, MON 89034 and TC1507 × MON 810 events have demonstrated excellent control of ACB over the 18 years of cultivation (Afidchao et al. 2013; Thompson et al. 2010; Caasi-Lit et al. 2018). Maize in the Philippines is planted on about 2.5 million ha, of which *Bt* maize hybrids occupied close to 0.66 million in 2019–20 (PSA 2020; USDA-GAIN 2021). GM maize (including herbicide-tolerant traits stacked with *Bt* traits) reached a peak of 0.730 million ha during 2012 and are planted on 0.6–0.7 million ha every year (USDA-GAIN 2020a). The high adoption of *Bt* maize in the Philippines has led to significant socio-economic benefits (Yorobe and Quicoy 2006; Yorobe and Smale 2012; Gonzales et al. 2013; Afidchao et al. 2013). FAW was first reported in the Philippines in 2019 (Navasero et al. 2019) and was reported to infest an area of 8,000 ha, mostly conventional maize, as of June 2020 (https://www.da.gov.ph/da-allots-p150m-to-help-farmers-control-fall-armyworm/). One possible reason for low infestations and delayed spread could be the adoption and cultivation of *Bt* maize (USDA-GAIN 2020a). There are no more recent estimates from the Philippines of FAW infestations.

**Table 3:** *Bt m*aize events approved for cultivation in the Philippines and Vietnam (Sources: Bureau of Plant Industry, Philippines and Ministry of Natural Resources and Environment (MONRE), Vietnam)

Technology Provider	*Bt* maize event	*Bt* gene(s) expressed	Country—year of approval for cultivation
Bayer Crop Science	MON 810	*cry1Ab*	Philippines—2002
	MON 89034	*cry1A.105* + *cry2Ab2*	Philippines—2010, Vietnam—2014
Pioneer Hi-bred	TC1507	*cry1F*	Philippines—2013, Vietnam—2016
Syngenta	Bt11	*cry1Ab*	Philippines—2005, Vietnam—2015
	MIR162	*vip3Aa20*	Philippines—2018

**Fig. 3:**
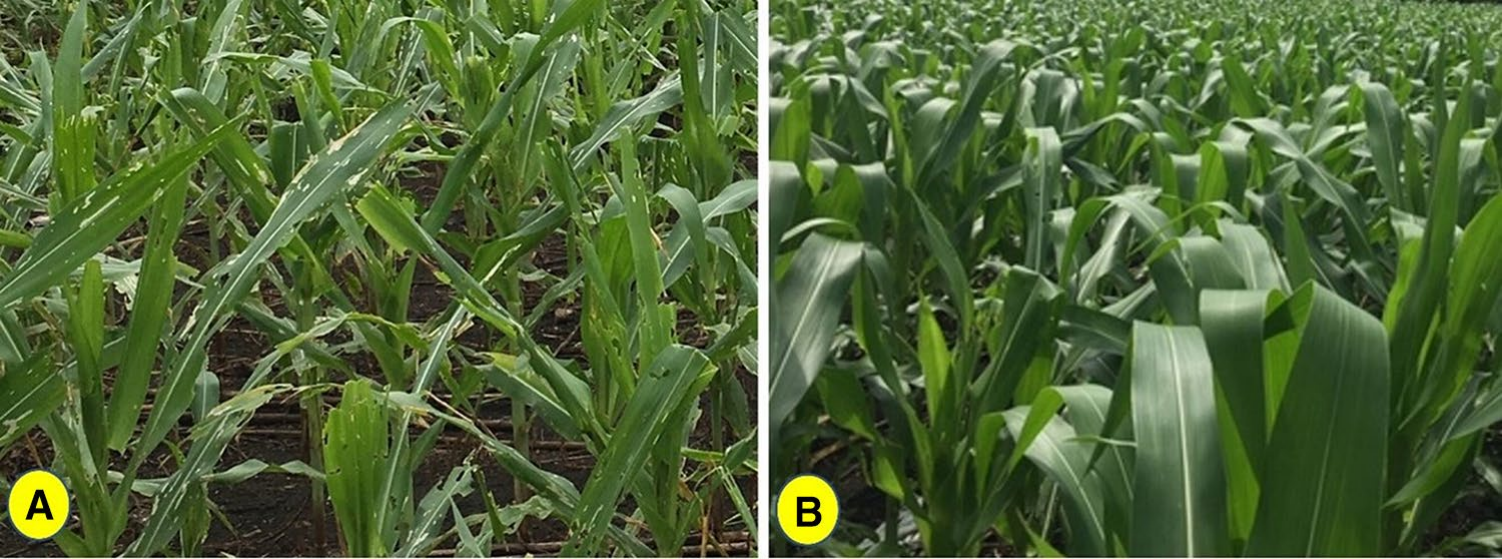
Photographs from a field trial in Vietnam showing **A** FAW-damaged conventional (non-*Bt*) maize hybrid with extensive foliar damage and **B** *Bt* maize (MON 89034) hybrid expressing the Cry1A.105 and Cry2Ab2 proteins with no FAW damage. Note: *Bt* maize was planted as a blend containing 5% non-*Bt* seed ( *Source*: Bayer Crop Science, Vietnam)

The evaluation of *Bt* maize in Vietnam began in 2010 and since then three *Bt* maize events (MON 89034, Bt11 and TC1507) have been approved for environmental release (Table 3). Based on the biosafety approval given by MONRE, the Ministry of Agriculture and Rural Development approved *B*t maize hybrids containing the MON 89034  (Fig. 3) and Bt11 events. In 2015, when they were first planted, *Bt* maize events occupied 3,500 ha and in 2019 they covered 92,000 ha, which was 10.2% of the total maize area (USDA-GAIN 2020b; Brookes and Dinh 2021). Brookes and Dinh (2021) highlighted the economic and environmental benefits of GM maize cultivation in Vietnam. FAW was documented in Vietnam in 2019 and was reported to have affected 35,000 ha in the country. However, in 2020, FAW infestations were reduced in earlier affected areas and this may be related to an increase in planting of *Bt* maize hybrids (USDA-GAIN 2020c).

In Pakistan, conventional maize was grown on 1.413 million hectares in 2019, with a consistent increase in area and production over the last 10 years (FAOSTAT 2021). Maize stem borer is the primary pest of maize (Arabjafari and Jalali 2007) in Pakistan, followed by armyworm species (*Spodoptera* spp., *Mythimna* spp.) and shoot fly (*Atherigona soccata*) which can reduce yield by 10–30% (Naz et al. 2003; Ahmed et al. 2003). FAW was first reported in 2019–20 in the maize growing districts of Sindh and Faisalabad, with Infestation levels of up to 80% (Naeem-Ullah et al. 2019; Gilal et al. 2020; Khan et al. 2020). Considering the management challenges with key maize pests, the evaluation of *Bt* maize events started in 2009. After seven years of evaluation, in 2016, the National Biosafety Committee of the Ministry of Climate Change approved stacked GM maize expressing insect resistance and herbicide tolerance traits for commercial cultivation. The GM maize products approved include stacked events with MON 810, MON 89034, TC1507 and TC1507 × MON 810. However, the varietal registration of the above-mentioned GM hybrids is pending approvals by the Federal Seed Certification and Registration Department, Ministry of National Food Security and Research. Other stacked events being tested contain insect-resistant combinations Bt11 × MIR162, Bt11 × MIR162 × MON 89034 and MON 89034 × TC1507.

Maize is planted over approximately 40 million ha in China (FAOSTAT 2021). The major lepidopteran pests of maize are ACB and CEW, causing 10–30% yield losses every year (Wang et al. 2000, 2021; Shen et al. 2016; Liu et al. 2016) with ACB alone causing an estimated 6–9 million tons of lost yield annually (He et al. 2003). Adding to these production constraints, FAW was first documented in China in 2019 (Sun et al. 2021a; Wu et al. 2019) and was observed in all maize-growing regions of China by mid-2019 (Jiang et al. 2019; NATESC 2019; Yang et al. 2021). By late 2019, FAW had damaged a total of 1.08 million ha of crops, mainly maize (Jiang et al. 2019). Qin et al. (2020) estimated that the potential economic losses to maize by FAW in China could be at least US$5.8 billion annually, of which US$3.95 billion can be managed with effective control measures in place. Most of the *Bt* maize events developed in China express Cry1 and/or Cry2 class proteins, as reviewed by Liu et al. (2016). These include events expressing single genes e.g., Cry1Ab (Wang et al. 2015) and fusion proteins such as Cry1Ab/Vip3A (Chang et al. 2013a, b) and Cry1Ah/Cry1Ie (Yang et al. 2012). All these events demonstrated efficacy against ACB under laboratory and field conditions. In a recent published study by Sun et al. (2021b), three *Bt* maize events—Ruifeng 125 (expressing Cry1Ab/Cry2Aj), DBN9936 & DBN9978 (expressing Cry1Ab)—were evaluated and found to be highly efficacious against ACB, including a Cry1C-resistant strain (Sun et al. 2021b). The invasion of FAW in 2019 triggered evaluations of available and new *Bt* maize events across China. FAW populations that invaded China were found to be susceptible to commonly used *Bt* proteins, e.g., Cry1, Cry2 and Vip3 classes (Li et al. 2019, 2020), and maize events, e.g., DBN9936 (expressing Cry1Ab protein) and the pyramided events, e.g., DBN3608 and DBN3601 (expressing Cry1Ab + Vip3A proteins) (Zhang and Wu 2019). There are three *Bt* maize event approvals (biosafety certificates) given by MARA in China since 2020 (Table 4), but no *Bt* maize hybrid approvals have been given yet.

**Table 4:** List of GM maize events offering control of lepidopteran insects that have been approved by the Ministry of Agriculture and Rural Affairs (MARA) in China

Event name	Genes	Targeted insects	Technology developer
DBN9501	*vip3Aa19, pat*	Fall armyworm	Beijing Da Bei Nong Biotech Ltd
DBN9936	*cry1Ab, epsps*	Asian corn borer	Beijing Da Bei Nong Biotech Ltd
Ruifeng125	*cry1Ab/cry2Aj, g10evo-epsps*	Asian corn borer	Hangzhou Ruifeng Biotech Ltd and Zhejiang University

*Source*: MARA, China

### Additional countries with potential for cultivation of *Bt* maize to manage FAW in Asia

India and Indonesia are the two largest maize-growing countries after China in Asia. Maize reached a peak of 8.7–9.9 million ha during 2018–19 in India, while in Indonesia an area of 4.5–5.7 million ha was planted in the past few years (FAOSTAT 2021). In both India and Indonesia, lepidopteran pest pressure, including from FAW, is high with heavy economic impacts and *Bt* maize should be considered as part of the IPM toolbox. However, no *Bt* maize events have been approved in either country.

In India, the stem borers, *C. partellus* and *Sesamia inferens* (Walker), are the major pests limiting maize production (Kumar et al. 2013). *C. partellus* alone was reported to cause yield losses up to 80% in some regions (Singh and Sajjan 1982; Chatterji et al. 1969). FAW was first detected in India in mid-2018, when Ganiger et al. (2018) reported it in southern India. By August 2019, FAW was reported from all maize-growing states (Sharanabasappa et. al. 2018; ICAR-NBAIR, 2018; Bhavani et al. 2019; Suby et al. 2020; Sharma 2021). Balla et al. (2019), using rainfall patterns, remote sensing and imaging, predicted yield losses from FAW infestation could be up to 33%.

In Indonesia, the major production constraint for maize is insect pests including ACB and CEW (Swastika et al. 2004). ACB has been the primary pest and causes significant damage to maize plants (Trisyono et al. 2020). Subiadi et al. (2014) reported 3.76–4.94% yield reduction per ACB larva depending on crop growth stage, with total yield loss exceeding 20% in the worst-case scenarios. In the last two years, FAW has gained economic importance after being first documented in mid-2019. The first report of FAW occurrence observed FAW on 100% of maize plants in the Lampung area, Sumatra (Trisyono et al. 2019). Supartha et al. (2021) surveyed FAW infestations in Bali province and reported that infestations began in the 2^nd^ week of planting (19.12%), peaked at 4^th^ week of planting (66.41%) and remained above 10% until 8^th^ week. This indicates the continued presence of FAW and the potential impact on maize yields.

## Field-evolved *Bt* resistance in FAW populations

The primary threat to the sustainable use of *Bt* maize technologies is the evolution of insect resistance. The first case of documented field-evolved resistance to *Bt* maize in FAW was for Cry1F-based maize hybrids in Puerto Rico (Storer et al. 2010, 2012a). Several factors apparently drove the evolution of FAW resistance in Puerto Rico, including the island setting limiting insect migration and the tropical climate conducive to year-round cultivation of maize (Storer et al. 2010). Subsequently, field resistance to Cry1F maize was also detected in the southeastern USA (Niu et al. 2013; Huang et al. 2014) and in the Brazilian state of Bahia, in the latter case approximately three years after first commercial plantings (Farias et al. 2014a, b). Low compliance with IRM recommendations seems to be a major cause of the rapid onset of resistance to Cry1F in FAW in Brazil (Farias et al. 2014b). Bernardi et al. (2015a) also detected partial cross-resistance among Cry1 proteins in FAW, meaning that the Cry1F resistance conferred some resistance to Cry1A.105 and Cry1Ab. However, no significant cross-resistance was found between Cry1F and Cry2Ab2. The potential cross-resistance between Cry1F, Cry1A.105 and Cry1Ab in FAW was previously shown through in vitro assays by Hernández-Rodríguez et al. (2013). Omoto et al. (2016) documented the evolution of field-relevant resistance in FAW to Cry1Ab in Brazil, potentially due to either selection from the use of MON 810 and/or cross-resistance to Cry1F.

The use of *Bt* maize hybrids with less-than-ideal IRM fit (e.g., less-than-high-dose technologies, components of *Bt* pyramids with cross-resistance to other *Bt* proteins in the landscape) combined with low compliance with IRM recommendations seems to be a common theme across the FAW resistance cases in South America (Farias et al. 2014a; Chandrasena et al. 2017). The deployment of MIR162-based maize technologies (expressing Vip3Aa20) carrying an effective new mode of action to counter FAW resistance to *Bt* maize technologies in South America has been an important addition to the “toolbox” for FAW control. However, poor compliance with IRM recommendation was a critical contributor to the observed cases of FAW resistance to *Bt* proteins in South America (Farias et al. 2014a; Chandrasena et al. 2017) and continues to be a threat to the sustainability of *Bt* maize technologies in the region.

## Insect resistance management (IRM) for *Bt* maize

The proactive implementation of effective IRM plans can delay resistance evolution to *Bt* maize in FAW populations. The central components of an IRM strategy for *Bt* crops are the “dose” of the *Bt* technology and ensuring that the “refuge” is present refuge needs of the product are met, typically via the planting of structure refuges. The dose of a *Bt* technology refers to the level of control of susceptible and heterozygous-resistant insects; ideally, a *Bt* technology should control nearly all heterozygotes in the pest population of the pest and thereby eliminate most of the resistance alleles from the target pest population (Gould 1998). A refuge ensures that a sufficient population of susceptible insects is available to mate with the few homozygous-resistant insects that may survive in *Bt* maize fields. The combination of high-dose products to control heterozygous individuals and sufficient refuge can significantly dilute the frequency of resistance alleles in the insect population over time, thereby delaying the evolution of insect resistance (Gould 1998; Tabashnik and Carrière 2017; Van den Berg et al. 2021).

Therefore, an effective IRM plan should include:

1. Removal of resistance alleles/genes from the insect population through *Bt* technologies with effective dose or high-dose expression of Bt proteins
2. Sufficient refuge for *Bt*-susceptible target insects
3. Deploy *Bt* technologies expressing two or more Bt proteins with independent modes of action against the target insect species
4. Educate and train farmers and relevant stakeholders on practices to manage resistance
5. Monitor the development of insect resistance

Although MIR162 expressing the Vip3Aa20 protein is currently the most effective *Bt* gene maize event, i.e., has the highest measured dose against FAW larvae (Bernardi et al. 2015b), the latest generations of *Bt* maize combining at least two Bt proteins active against FAW and representing unique and independent modes of action characterize a robust alternative to manage this species (Horikoshi et al. 2016, 2021; Moscardini et al. 2020; Tavares et al. 2021). These products, known as *Bt* pyramids, combine robust protection against larval feeding injury of FAW (Waquil et al. 2013; Moscardini et al. 2020) and improved IRM value (Roush 1998; Storer et al. 2012b). *Bt* maize products with two or more effective *Bt* proteins with distinct modes of action significantly improve the likelihood of delaying resistance; however, sustaining reasonable compliance with structured refuge recommendations is also essential for sustained management of FAW. Failure to maintain adequate compliance with structure refuge recommendations was one of the main causes for FAW resistance to *Bt* maize technologies in South America. Among the reasons for the low levels of compliance are the economic and opportunity costs of refuge plantings. The use of seed mixtures of *Bt* and non-*Bt* seeds in the same package can transfer the responsibility of ensuring compliance to manufacturers instead of relying on farmers to plant a structured refuge (Carroll et al. 2012). Despite the benefits of seed mixtures in ensuring refuges are present within Bt maize fields, larval movement between *Bt* and non-*Bt* plants in a seed mix field can increase the risk of resistance evolution. FAW larvae not receiving a lethal dose that move off *Bt* plants onto adjacent non-*Bt* plants, and movement of larger and less susceptible larvae from non-*Bt* to adjoining *Bt* plants, could increase heterozygote fitness and intensify selection for resistance (Carroll et al. 2012). In addition, movement of susceptible FAW larvae off non-*Bt* plants onto neighboring *Bt* plants reduces refuge effectiveness by reducing the number of susceptible insects produced by refuge plants (Carroll et al. 2012). Although FAW larvae are highly mobile (Pannuti et al. 2016; Malaquias et al. 2017; Sokame et al. 2020), the use of effective *Bt* maize pyramids can reduce these risks associated with seed mix refuges for FAW (Bernardi et al. 2015b; Dimase et al. 2021; Horikoshi et al. 2021; Tavares et al. 2021).

Once the resistance risk of a *Bt* maize technology is properly assessed and a refuge recommendation is defined, it is fundamental to integrate the IRM recommendation into business plans and ensure resources are available to: a) implement training and education programs for farmers and other key stakeholders; b) track adoption and use patterns of a *Bt* maize technology and the level of compliance with structured refuge by farmers (if structured refuge is part of the IRM recommendation); c) monitor the development of resistance in populations of the target pest and d) follow up on the cases of unexpected injury to the technology and recommend remediation practices (Head and Greenplate 2012).

## Host plant resistance to FAW: critical gaps, challenges and priorities

### Critical gaps and challenges

- An array of FAW-tolerant/FAW-resistant germplasm in diverse genetic backgrounds needs to be developed and deployed in both Africa and Asia. A major obstacle to breeding crop varieties with FAW resistance using conventional breeding is the low frequency of resistant genotypes in germplasm collections. Therefore, it is imperative both to widen the search for sources of native genetic resistance to FAW and to discover, validate and ultimately deploy genomic regions conferring resistance to FAW using either marker-assisted breeding or genomic selection, as appropriate, depending on presence/absence of major haplotypes conferring resistance to FAW.
- It must be noted that farming communities need elite crop varieties with not only FAW tolerance/resistance, but also a package of other traits relevant for that specific agroecology or market segment, including high yield, abiotic stress tolerance, disease resistance, nutrient and water use efficiency, nutritional enhancement, etc. Often the sources of genetic resistance to FAW may not be directly useful as elite parental lines of commercial hybrids/varieties. Therefore, intensive and accelerated breeding efforts are required to transfer native resistance from validated sources of resistance into diverse, Africa-adapted and Asia-adapted elite maize products (inbreds/hybrids/OPVs) for deployment to farming communities. Similar efforts are needed in other major crops, such as sorghum and millets, affected by FAW in Africa and Asia.
- Lack of adequate investment in accelerated and intensive breeding for native genetic resistance to FAW in Africa and Asia is hampering progress by the international agricultural research centers and national partners to come out with solutions for FAW management based on host plant resistance.
- Another important gap that needs urgent attention is the stacking of transgenic insect-resistant traits with native genetic resistance and the combined value they could generate, ensuring sustainable yield protection from pests such as FAW.
- Deploying improved maize varieties with genetic resistance to FAW (native or transgenic) has significant potential to reduce the use of pesticides by farmers. Studies should be done to empirically quantify the reduction in pesticide use together with the increase in resilience and productivity that comes with deployment of host plant resistance.
- Functional and effective management of intellectual property seems to be a challenge in parts of Africa and Asia, likely impacting investments on transgenic research to address issues insect control in those regions. This would require policies in place to ensure there is return on the investments made by the private and or public sectors in developing these technologies.
- Regulatory systems that are restrictive for the research and cultivation of transgenic crops in countries in Africa and Asia are limiting farmers access to technologies to manage FAW. Consumer acceptance of transgenic crops and or limited technical capacity to evaluate safety of these products seems to be the main drivers of this bottleneck. These technologies have been proven safe to humans, non-target organisms and the environment. Therefore, it is critical to work across stakeholders to understand the causes and how to improve the functionality of the regulatory systems in Africa and Asia. Moreover, capacity building on biosafety may be necessary in several countries in Africa and Asia to enable the development of science-based and efficient regulatory frameworks to assess the safety of *Bt* crops in the region.

### Priorities

In terms of native genetic resistance to FAW, the key priorities are:

1. Varietal release and widespread deployment of “first-generation” white maize hybrids with FAW resistance, developed recently by CIMMYT and now available to partners, especially in SSA; these hybrids can also be potentially tested in Asian countries where white maize varieties are grown and consumed by local populations.
2. Fast-tracked introgression of sources of native genetic resistance to FAW into Africa-and Asia-adapted germplasm, and release of next-generation products with native genetic resistance to FAW in Africa and Asia.
3. Discovery/validation of genomic regions for resistance to FAW in maize using appropriate populations and exploring the possibility of genomic prediction for developing novel Africa-adapted/Asia-adapted FAW-tolerant/FAW-resistant maize varieties.
4. Strengthening the capacity of NARES institutions in Africa and Asia in breeding and deploying improved maize varieties with resistance to FAW and other important adaptive and agronomic traits relevant for the smallholders.

Regarding transgenic resistance to FAW, the major priorities are:

1. Accelerated testing and deployment of *Bt* maize with proven efficacy, biosafety and environmental safety with appropriate support from policy makers and regulatory authorities.
2. Pyramiding transgenes with different modes of action (*e.g.*, *cry* + *vip* genes), instead of single-gene deployment, as a part of IRM strategy.
3. Implementing IRM and proper stewardship wherever *Bt* maize varieties have been deployed in Africa and Asia, to ensure sustainable protection against the pest.

## Conclusion

Sustainable control of FAW is best achieved when farmers use host plant resistance as part of an IPM strategy, together with good agricultural practices, pest scouting, biological control, agro-ecological management and judicious use of environmentally safer pesticides. Intensive efforts are being made in Africa by CIMMYT and partners to identify, validate and develop elite maize germplasm with native genetic resistance to FAW. These efforts need to be further accelerated and intensified in both Africa and Asia to derive elite tropical/subtropical germplasm suitable for different agro-ecologies and market segments. Such products must combine FAW resistance with other desirable and relevant traits for resource-constrained smallholder farmers in the target geographies.

In addition, *Bt* maize hybrids carrying lepidopteran-specific transgene(s), wherever released in Africa or Asia, can become an important tool in the IPM toolbox for FAW management. Bringing the benefits of *Bt*-based solutions for FAW management more extensively into Africa and Asia would, however, require overcoming existing regulatory, political and consumer acceptance hurdles. In countries where *Bt* maize is already commercialized, it is important to devise and implement effective IRM strategies. Combinations of native genetic resistance and *Bt* maize also need to be explored as a path to more effective and sustainable host plant resistance options.

## Data Availability

No datasets were specifically generated for this publication. Pedigree and characterization of CIMMYT maize lines, including lines with native genetic resistance to FAW, are available at CIMMYT Dataverse (https://data.cimmyt.org/file.xhtml?persistentId=hdl:11529/10246/23&version=14.0). The sources of CIMMYT datasets used for Kamweru et al. (2022) are available at: http://data.cimmyt.org.

## Abbreviations

ARIs: Advanced Research Institutes
AATF: African Agricultural Technology Foundation
*Bt*: *Bacillus thuringiensis*
CML: CIMMYT Maize Line
DH: Doubled haploid
FAW: Fall armyworm
GM: Genetically modified
GWAS: Genome-wide association study
GEM: Germplasm enhancement of maize
IRMA: Insect Resistant Management for Africa
IPM: Integrated pest management
IRM: Insect resistance management
JA: Jasmonic acid
KALRO: Kenya Agricultural and Livestock Research Organization
Mb: Megabase
MIRT: Multiple insect-resistant tropical
MBR: Multiple borer resistant
NARES: National Agricultural Research & Extension System
OPVs: Open-pollinated varieties
QTL: Quantitative trait loci
SSR: Simple sequence repeat
SNP: Single-nucleotide polymorphism
STMA: Standard Material Transfer Agreement
SSA: Sub-Saharan Africa
USDA-ARS: United States Department of Agriculture-Agricultural Research Service
VIP: Vegetative insecticidal protein

## Footnotes

1 Seed of the FAW-tolerant CMLs can be sourced from CIMMYT Genebank at Mexico under a Standard Material Transfer Agreement (SMTA).
